# Pearl Powder Hybrid Bioactive Scaffolds from Microfluidic 3D Printing for Bone Regeneration

**DOI:** 10.1002/advs.202304190

**Published:** 2023-10-23

**Authors:** Lei Yang, Lu Fan, Xiang Lin, Yunru Yu, Yuanjin Zhao

**Affiliations:** ^1^ Department of Rheumatology and Immunology, Nanjing Drum Tower Hospital, School of Biological Science and Medical Engineering Southeast University Nanjing 210096 China; ^2^ Oujiang Laboratory (Zhejiang Lab for Regenerative Medicine, Vision and Brain Health), Wenzhou Institute University of Chinese Academy of Sciences Wenzhou 325001 China

**Keywords:** 3D printing, bone regeneration, growth factor, microfluidics, pearl powder

## Abstract

The development of bioactive scaffolds by mimicking bone tissue extracellular matrix is promising for bone regeneration. Herein, inspired by the bone tissue composition, a novel pearl powder (PP) hybrid fish gelatin methacrylate (GelMa) hydrogel scaffold loaded with vascular endothelial growth factor (VEGF) for bone regeneration is presented. With the help of microfluidic‐assisted 3D printing technology, the composition and structure of the hybrid scaffold can be accurately controlled to meet clinical requirements. The combination of fish skin GelMa and PP also endowed the hybrid scaffold with good biocompatibility, cell adhesion, and osteogenic differentiation ability. Moreover, the controlled release of VEGF enables the scaffold to promote angiogenesis. Thus, the bone regeneration in the proposed scaffolds could be accelerated under the synergic effect of osteogenesis and angiogenesis, which has been proved in the rat skull defect model. These features indicate that the PP hybrid scaffolds will be an ideal candidate for bone regeneration in clinical applications.

## Graphical abstract

A pearl powder hybrid bioactive scaffolds from microfluidic 3D printing is developed for bone regeneration. The pearl powder scaffold with good biocompatibility, cell adhesion, and osteogenic differentiation ability, which can load with vascular endothelial growth factor to promote angiogenesis. Animal test indicates that this scaffold can effectively promote bone regeneration by coordinating angiogenesis and osteogenesis.

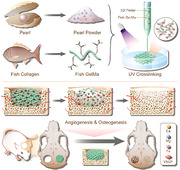

## 1 Introduction

The bone defect caused by trauma, infection, tumor, and osteomyelitis is a common orthopedic disease.^[^ ^1^, ^2^ ^]^ Especially, large bone defects, where defected area exceeds the critical state for autologous repair, cannot heal spontaneously with organic stimulation and could result in bone resorption, osteogenic nonunion, and delayed bone healing.^[^ ^3^, ^4^ ^]^ To facilitate bone regeneration, orthopedic surgical treatment is often necessary. The most commonly used approach in orthopedic surgery is bone grafting, which involves autologous and allogeneic bone grafts.^[^ ^5^, ^6^ ^]^ However, bone grafting still poses the risk of bone source shortage, potential immune rejection, and viral transmission.^[^ ^7^, ^8^ ^]^ As an alternative, tissue engineering scaffolds based on different materials such as hydroxyapatite (HAP),^[^ ^9^, ^10^ ^]^ bioactive glass (BS),^[^ ^11^, ^12^, ^13^ ^]^ sodium alginate (ALG),^[^ ^14^, ^15^ ^]^ and gelatin methacrylate (GelMa),^[^ ^16^, ^17^ ^]^ have been widely used for bone regeneration. Although these scaffolds have been effective in supporting cell migration and showed excellent osteoconductivity for bone repair, their biological activity is usually limited, and their composition significantly differs from that of natural bone tissue. In addition, poor integration with bone tissues would cause joint failure and reduce the therapy efficacy. Therefore, there is still a need for bioactive bone tissue‐mimicking scaffolds that exhibit satisfactory biological activity and excellent integration with bone tissues to meet the demands of bone regeneration.

**Figure 1:**
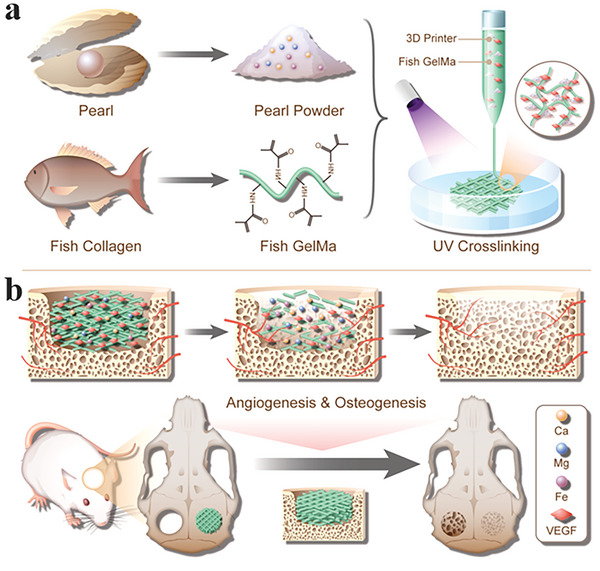
Schematic diagram of PP hybrid bioactive scaffold from microfluidic 3D printing for bone regeneration. a) The composition and microfluidic 3D printing of PP hybrid bioactive scaffold. b) The application of PP hybrid bioactive scaffold in bone regeneration.

Herein, inspired by the composition of natural mineralized products, which are usually with similar components of bone tissues, we present a bioactive pearl powder (PP) scaffold by microfluidic‐assisted 3D printing for bone regeneration, as schemed in **Figure** **1**. PP is a well‐known traditional Chinese medicine derived from the pearl. It has been applied in medical cosmetology, food additives, and especially bone tissue engineering for its superior biocompatibility, antioxidant, and osteogenic activity.^[^ ^18^, ^19^ ^]^ Fish skin GelMa from aquatic sources is widely available and inexpensive compared to porcine skin GelMa from terrestrial animal sources, which also poses unique functional properties.^[^ ^20^, ^21^ ^]^ Microfluidics is a promising technique in the fabrication of microfibers for varied applications due to its wide fluid compatibility and precise control over flow composition as well as flow rates, which have been used in drug release, cell culture and biosensor.^[^ ^22^, ^23^ ^]^ As an advanced additive manufacturing technique, 3D printing allows for the personalized design of implantable scaffolds to meet specific clinical needs.^[^ ^24^, ^25^ ^]^ Taking advantage of these, the integration of microfluidics and 3D printing would realize the yield of designable scaffolds for bone regeneration. However, the combination of PP and fish skin GelMa as a bioink in the microfluidic‐assisted 3D printing for bone engineering scaffold remains unexplored, and their biological activity in bone regeneration still needs investigation. In addition, bone healing bone healing is a complex and dynamic biological process that involves early‐stage angiogenesis and later‐stage osteogenesis. Supplementation of angiogenic substances, such as vascular endothelial growth factor (VEGF, known for its angiogenesis), in the early stage of bone repair can effectively promote vascular reconstruction and improve the transport of oxygen and nutrients, thus recruiting endogenous stem cells to reconstruct the bone tissue.^[^ ^26^ ^]^ Accordingly, in the late stage of bone repair, supplementation of osteogenic differentiation substances to promote the conversion of stem cells to osteoblasts is the key to the treatment of bone defects.^[^ ^26^ ^]^ Therefore, combining the angiogenic effect of VEGF and the osteogenic effect of PP to develop a novel scaffold that can effectively promote bone repair is highly desirable.

In this paper, we employed the microfluidic 3D printing technology to develop PP hybrid hydrogel scaffolds loading with VEGF for bone regeneration (Figure 1). During this process, the mixture of PP, fish skin GelMa, and VEGF was used as the bioink, and the scaffold was achieved by a microfluidic‐assisted printing method coupled with in situ photopolymerization. The high controllability of microfluidic 3D printing imparted the scaffolds with adjustable shapes for different application purposes; the in situ photopolymerization well retained the scaffold structure, realized the encapsulation of bioactive additives, and maintained their bioactivity. Owing to the intrinsic properties of PP and fish skin GelMa, the resultant hybrid bioactive scaffold showed excellent biocompatibility, cell adhesion, and osteogenic differentiation. The loading and controllable release of VEGF from the PP scaffold can promote vascular reconstruction of the damaged area, thus improving the transport of oxygen and nutrients in the early stages of bone repair. Subsequently, with the degradation of the PP scaffolds in the late stage of bone repair, the release of osteogenic substances of the PP scaffolds can effectively promote the differentiation of endogenous stem cells into osteoblasts, thus promoting bone repair. Taken together, this composite scaffold exhibits both the osteogenic effect of PP and the angiogenic effect of VEGF, which was further proved by in vivo bone regeneration models. These features indicate that the PP hybrid bioactive scaffolds loaded with VEGF are adaptive in large bone defects, and can be an ideal candidate for clinical bone regeneration.

## 2 Results and Discussion

### 2.1 Characterization of the Scaffold

**Figure 2:**
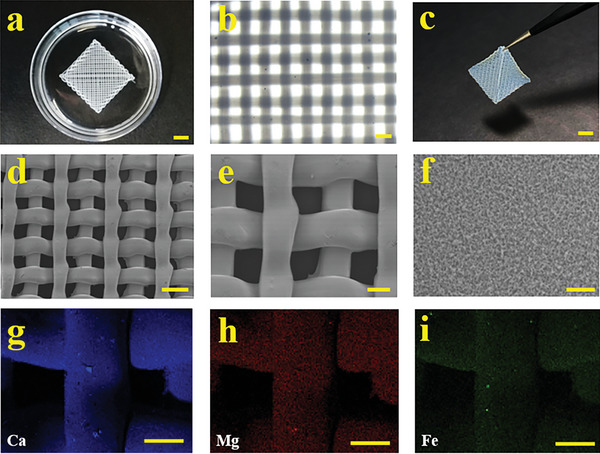
Characterization of PP scaffold. a) The digital image of the PP hybrid scaffold. b) The microscopic image of the scaffold showing its stacked architecture. c) Digital image of the free‐standing dehydrated PP hybrid scaffold. d–f) SEM images of (d) the PP Scaffold, (e) the fiber stack, and (f) the surface of the PP scaffold. g–i) Energy dispersion spectroscopy analysis showing (g) Ca, (h) Mg, and (i) Fe elements of the PP scaffold. Scale bars in (a‐c) are 5, 1, 5 mm, respectively. Scale bars in (d‐e) are 500, 200, and 10 µm, respectively. Scale bars in (g‐i) are 200 µm.

The bioink was a mixture of 25% GelMa, 2% PP and 0.5% LAP. The bioink was first printed out from a capillary nozzle, forming designed shapes, including square, triangle, and circle, and solidified under UV irradiation (**Figure** **2**a; Figure S1a, Supporting Information). It could be seen that the uniform morphology of fiber was well reserved along with the stacked 3D architecture of the scaffold (Figure 2b,c). This could be attributed to the matched phase flow and nozzle moving rates during printing, as well as the rapid polymerization of the stream under UV light during collection. The influence of the PP addition on the formation of the scaffold was also investigated. In detail, the scaffold was with solid and distinct structure when the concentration of PP was in the range of 0%–2%, while the fiber of the scaffold began to fuse when the concentration increased to 3%–4% (Figure S1b, Supporting Information). It is possible that the high PP concentration affects the polymerization rate of the GelMa and reduces the printing accuracy. We also tested the effect of PP addition on the mechanical properties of the scaffold. The results showed that the 0% PP addition had the lowest compression modulus and showed multiple fractures. The 2% and 4% PP addition had similar fracture pressures, but the 2% PP addition had greater maximum compression than 0% PP and 4% PP (Figure S2, Supporting Information). The microstructure of our scaffold was observed by scanning electron microscopy (SEM) (Figure 2d‐f). It also demonstrated the porous surface of the PP scaffold, which could facilitate the exchange of nutrients and oxygen as well as the release of bioactive additions (Figure 2e,f). The energy dispersion spectroscopy (EDS) results showed that trace elements of Ca, Mg, and Fe, coming from PP, exhibited in the scaffold, which revealed the successful loading of the osteogenic composition of PP (Figure 2g–i; Figure S3, Supporting Information).

### 2.2 In Vitro Biocompatibility and Angiogenesis of the Scaffold

**Figure 3:**
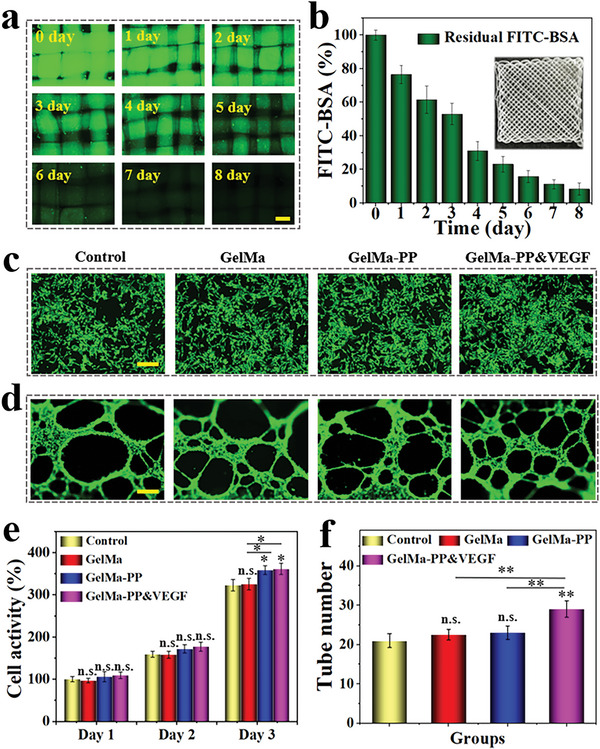
Drug release, and in vitro biocompatibility and angiogenesis studies. a) Fluorescent images of FITC‐BSA laden GelMa‐PP scaffold in PBS from 0 to 8 days. b) The fluorescent intensity of the scaffold. c) Calcein‐AM staining of BMSCs after 3 days of co‐incubation with different groups. d) Tube formation of HUVECs in different groups after cultivation for 6 h. e) Statistical analysis of cell activity of BMSCs in different groups from day 1 to day 3. f) Statistical analysis of the formed tubes in different groups after 6 h of co‐culture. Scale bars are 200 µm in (a), (c), and (d). Data (n≥3) are shown as mean ± SD. n.s.: no significant, *p< 0.05, **p< 0.01, ***p< 0.001.

The loading of VEGF would impart the PP scaffold with angiogenic ability. Before investigating this angiogenic ability, the drug loading and release were studied at in vitro level. Specifically, the fluorescent isothiocyanate bovine serum albumin (FITC‐BSA) was chosen as a model drug and encapsulated into the scaffold, after which the scaffold was immersed in the phosphate‐buffered saline (PBS) solution and the fluorescence of the scaffold was observed and recorded. The uniform and bright fluorescence of the scaffold shown in **Figure** **3**a indicated the successful encapsulation of FITC‐BSA; the sustainably reduced fluorescence intensity suggested the slow release of the drug (Figure 3b). The function of the scaffold is also related to its biodegradation; thus, the biodegradability of the PP scaffolds was investigated here. The results indicated that the scaffold experienced a slow degradation period in the early two weeks and the entire degradation period lasted almost 5 weeks (Figure S4, Supporting Information).

The biocompatibility was also investigated by co‐culturing the scaffolds with bone marrow mesenchymal stem cells (BMSCs), and osteoblastic MC3T3‐E1, respectively. It could be seen that after 3 days of cultivation, both BMSCs and MC3T3‐E1 in the scaffold group showed ideal cell densities and good morphologies compared with the control group (Figure 3c; Figure S5a, Supporting Information). Quantitively, the statistical CCK‐8 results showed that the cell activity of BMSCs in the PP hybrid scaffold group was better than other groups, while that of MC3T3‐E1 in the PP hybrid scaffold had no obvious difference (Figure 3e; Figure S5b, Supporting Information). This may be because the trace elements from PP, such as the Mg,^[^ ^27^ ^]^ and Ca,^[^ ^28^ ^]^ can promote BMSCs cell proliferation while having limited effect on MC3T3‐E1.^[^ ^29^ ^]^ Besides the cytotoxicity evaluation, the blood compatibility of the scaffolds was also evaluated. The hemolysis results showed no obvious hemolysis of rat erythrocytes in the scaffolds groups, indicating that the resultant scaffold had excellent blood compatibility (Figure S6, Supporting Information).

The cell adhesion ability of the scaffold with or without PP was also studied. It demonstrated that the MC3T3‐E1 cells could adhere to the surface of both GelMa and GelMa‐PP scaffolds after 24 h of cultivation, suggesting the addition of PP would not affect the cell adhesion property of the scaffold (Figure S7, Supporting Information). To confirm the angiogenesis effect of the PP hybrid scaffold loaded with VEGF, an in vitro tube formation assay was employed. After incubation with human umbilical vein endothelial cells (HUVECs) for 6 h, the tube formation could be observed. Comparatively, the number of the formed tubes in the PP hybrid bioactive scaffold group was greatly increased (Figure 3d,f). These results indicated that the PP hybrid scaffold with VEGF loading displayed good cytocompatibility and could promote angiogenesis, which would be beneficial for further bone regeneration.

### 2.3 In Vitro Osteogenic Activity and Cell Migration Induction of the Scaffold

**Figure 4:**
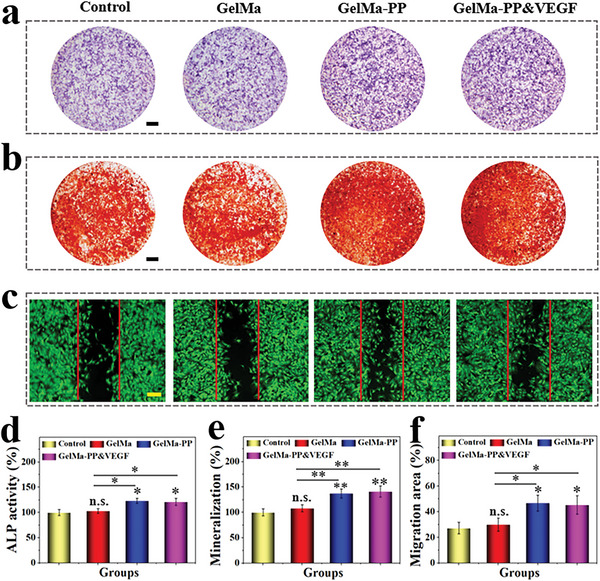
In vitro osteogenesis and cell migration test. a) ALP staining of BMSCs after two weeks of induction. b) AR staining of BMSCs after two weeks of incubation. c) Fluorescent images of BMSCs in different groups after 24 h of treatment. d–f) Quantitative analysis of (d) ALP activity, e) mineralization degree, and f) cell migration area. Scale bars are 2 mm in (a) and (b), and 150 µm in (c), respectively. Data (n≥3) are shown as mean ± SD. n.s.: no significant, * *p*< 0.05, **p< 0.01, ***p< 0.001.

To investigate the bone regenerative ability of the PP hybrid bioactive scaffold, BMSCs were cultured with different scaffolds including GelMa, GelMa‐PP, and GelMa‐PP&VEGF for two weeks. The impact of osteogenic induction was evaluated by using alkaline phosphatase (ALP) and alizarin red (AR) staining. The former suggests early bone formation, while the latter indicates the later calcium deposition during osteogenic induction. It demonstrated that the ALP activity was significantly improved by the PP‐containing groups, including GelMa‐PP and GelMa‐PP&VEGF groups (**Figure** **4**a,d). Furthermore, AR staining results indicated the deposition of calcium in all groups after two weeks of induction (Figure 4b). The quantification analysis further confirmed the enhanced calcium nodules in GelMa‐PP and GelMa‐PP&VEGF groups compared with the control and GelMa groups (Figure 4e). To confirm the osteogenic activity of PP hybrid bioactive scaffold, osteogenesis markers including the runt‐related transcription factor 2 (RUNX2), ALP, and osteocalcin (OCN) were further validated by western blots and RT‐qPCR (Figure S8, Supporting Information). The results showed that the osteogenic markers in the PP‐containing group were higher than those in the control and GelMa groups at both protein and mRNA levels. This could be ascribed to the Ca and Mg of PP, which could promote BMSCs proliferation, migration, and osteogenic differentiation.^[^ ^30^, ^31^ ^]^ In addition, some other components of PP, such as active proteins and glycoproteins, may be also conducive to osteogenesis.^[^ ^32^ ^]^ To verify our hypothesis, a cell scratch test was conducted. As shown in Figure 4c,f, the cell migration was obviously accelerated in GelMa‐PP and GelMa‐PP&VEGF groups than those in others. Altogether, the generated PP hybrid scaffold showed satisfactory in vitro osteogenic activity, which is beneficial for bone regeneration.

### 2.4 In Vivo Bone Regeneration Ability

**Figure 5:**
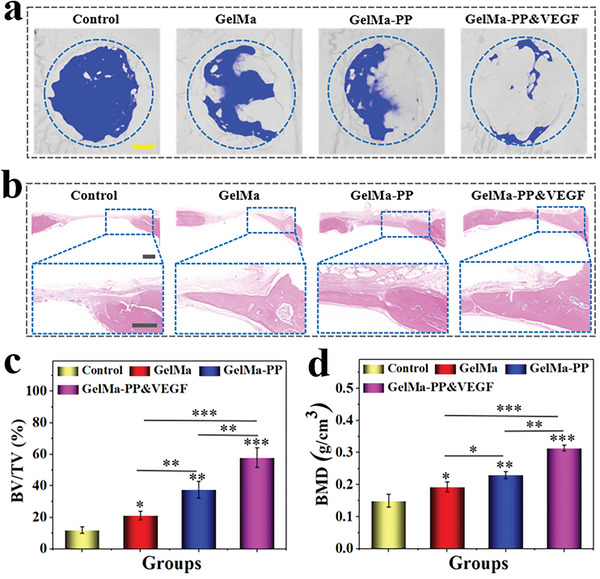
In vivo bone regeneration evaluation. a) Representative 3D reconstructed micro‐CT images of the rat skulls after 8 weeks of treatment. b) Representative H&E images of defected regions in different groups after 8 weeks of treatment. c,d) Quantitative analyses of (c) BV/TV and (d) BMD of newly‐formed bone tissues at defected sites. Scale bars are 1 mm in (a) and 500 µm in (b), respectively. Data (n≥3) are shown as mean ± SD. n.s.: no significant, *p< 0.05, **p< 0.01, ***p< 0.001.

Given the osteogenic PP and angiogenic VEGF of the hybrid scaffold, the in vivo bone regeneration application of the scaffolds was investigated using a rat skull defect model. After surgical operation, rats implanted with GelMa, GelMa‐PP, and GelMa‐PP&VEGF scaffolds or treated with PBS solutions were divided into GelMa, GelMa‐PP, GelMa‐PP&VEGF, and the control group, respectively. After eight weeks of therapy, the skulls were collected for micro‐CT monitoring and histological analysis as shown in **Figure** **5**a, where regenerated bone tissues were observed in the GelMa, GelMa‐PP, and GelMa‐PP&VEGF groups compared to the control group. Notably, the GelMa‐PP&VEGF group performed best in bone regeneration promotion. Hematoxylin‐eosin (H&E) and Masson's trichrome staining also confirmed the newly‐formed bone tissue in scaffold‐treated groups (Figure 5b; Figure S9, Supporting Information). Among them, the GelMa‐PP&VEGF group showed the best bone regeneration performance, which was consistent with the micro‐CT result, suggesting their superior capability in promoting bone repair. The quantitative analysis also revealed that the GelMa‐PP&VEGF group had the highest bone volume to tissue volume (BV/TV) and bone mineral density (BMD), showing an accelerated bone regeneration rate compared with other groups.

### 2.5 In Vivo Osteogenesis and Angiogenesis Capabilities

**Figure 6:**
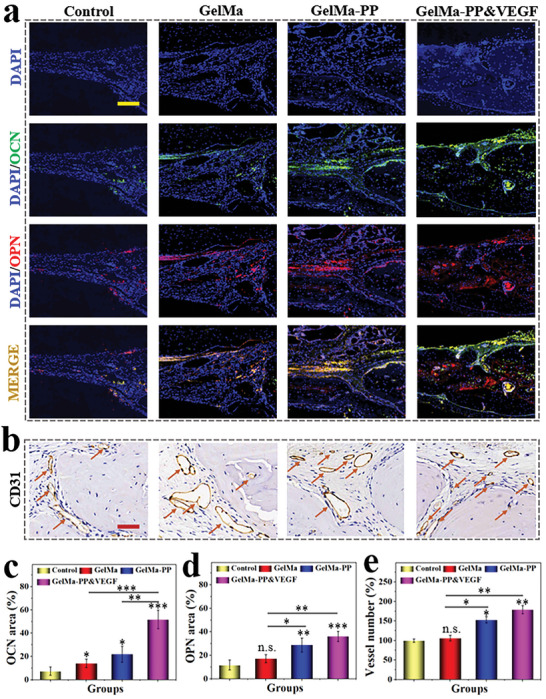
In vivo osteogenesis and angiogenesis evaluation. a) Immunofluorescent staining of DAPI, OCN, OPN, and the merged channel. b) Immunohistochemical staining of CD31 at the defect region. The arrows indicate the blood vessels. c,d) Quantitative analyses of (c) OCN and (d) OPN positive area percentage. e) Statistical results of vessel number at bone defect regions in different groups. Scale bars are 500 µm in (a) and 50 µm in (b). Data (n≥3) are shown as mean ± SD. n.s.: no significant, *p< 0.05, **p< 0.01, ***p< 0.001.

To further evaluate the osteogenic and angiogenic effects of PP hybrid bioactive scaffolds, the expression of osteopontin (OPN) as well as OCN (two markers of bone regeneration),^[^ ^33^, ^34^ ^]^ and platelet endothelial cell adhesion molecule‐1 (CD31, a marker of vascular endothelial cells)^[^ ^35^ ^]^ was studied by immunofluorescent and immunohistochemical staining, respectively. As shown in **Figure** **6**a, the expression of OPN and OCN positive protein was up‐regulated in GelMa‐PP and GelMa‐PP&VEGF groups compared with the other groups. Notably, the positive area percentage in the GelMa‐PP&VEGF group was significantly higher than others, indicating its best bone repair performance. Immunohistochemical staining also revealed more blood vessels in the GelMa‐PP&VEGF group than those in GelMa‐PP and GelMa groups, which proved that the released VEGF from PP hybrid bioactive scaffold could effectively promote revascularization and further promote bone regeneration (Figure 6b). Apart from osteogenic and angiogenic capabilities, the scaffold would not harm other organs, which could be manifested by HE staining of main organs (Figure S10, Supporting Information). All these results suggested that the PP hybrid bioactive scaffold loading with VEGF could effectively promote bone regeneration via a synergistic effect of osteogenesis and angiogenesis.

## 3 Conclusion

In conclusion, we presented a novel kind of PP hybrid bioactive scaffold from microfluidic 3D printing for bone regeneration. The scaffold consisting of PP, fish skin GelMa, and VEGF could be controllably obtained from the tailorable microfluidic printing technique. Benefitting from the mild but rapid photopolymerization, the VEGF was successfully loaded in the scaffold and maintained its high bioactivity. The encapsulation of PP equipped the scaffold with bone‐enhancing factors, calcium, magnesium, and iron; the fish skin GelMa matrix provided a good cell adhesion ability; the loading of VEGF enabled the scaffold to promote vascularization. All of these made the hybrid scaffold effectively up‐regulate the expression of ALP, OCN/OPN, and induce extracellular matrix mineralization, thus promoting osteogenic differentiation of stem cells both at in vitro and in vivo levels. These features indicated that our designed PP hybrid bioactive scaffold was promising in clinical bone regeneration treatment.

## 4 Experimental Section

### Materials and Animals

Lithium phenyl‐2,4,6‐trimethylbenzoylphosphinate (LAP), methacrylic anhydride, and fish gelatin were purchased from Sigma‐Aldrich (St. Louis, USA). GelMa was synthesized followed by a previous protocol.^[^ ^20^ ^]^ PP was obtained from Tongrentang (Beijing, China). VEGF, CCK8, FITC‐BSA, and staining kits of ALP and AR were obtained from Beyotime (Nanjing, China). Calcein‐AM/PI was purchased from Yanhui Biological (Shanghai, China). Matrigel matrix was obtained from BD Bioscience (Shanghai, China). The MC3T3‐E1 and HUVEC were purchased from YaJi Biological (Shanghai, China). The rat BMSCs and osteogenic induction medium were purchased from Cyagen Biosciences (Guangzhou, China). The antibodies of OCN, OPN, CD31, and 4′,6‐diamidino‐2‐phenylindole (DAPI) were purchased from Abcam (Cambridge, UK). The male SD rats at 8–10 weeks were obtained from Beijing Vital River Laboratory Animal Technology (Beijing, China). All animal experiments were approved by the Animal Care and Use Committee of Wenzhou Institute (WIUCAS22062001).

### PP Scaffold Preparation and Characterization

In general, 2 wt.% PP, 25 wt.% GelMa, and 0.5 wt.% LAP with 2 µg mL^−1^ VEGF were dissolved in ultrapure water as the precursor of the PP scaffold. Then the mixture was pumped into the microfluidic 3D printing nozzle at the flow rate of 2 mL h^−1^ to generate the PP scaffold. The scaffold was collected in PBS solution with 2 µg mL^−1^ VEGF and solidified under UV irradiation. Then, the morphology of the PP scaffold was observed under a microscope (JIANGNAN, JSZ6S). The microstructure of the PP scaffold was examined by scanning electron microscopy (HITACHI, S‐3000N). The compression test of the PP scaffold was examined by electronic universal testing machine(Instron,5944).

### In Vitro Drug Release Test

In order to examine the drug release of PP scaffolds, FITC‐BSA was encapsulated as a model drug. Generally, the mixture of 2 wt.% PP, 25 wt.% GelMa, 0.5 wt.% LAP, and 1 mg mL^−1^ FITC‐BSA was taken as the bioink to print the PP scaffold. After the FITC‐BSA loaded PP scaffold was cleansed by PBS 3 times, the PP scaffold was immersed in PBS on a shaker at 100 rpm at 37 °C. The fluorescence intensity of the PP scaffolds was observed and captured from 0 to 8 days under a fluorescence microscope (OLYMPUS IX71) to monitor the drug release.

### Biocompatibility Test

The primary P3 BMSCs from rats and the osteoblastic MC3T3‐E1 cell line were used to test the biocompatibility of scaffolds on stem cells and osteoblasts. The cells were co‐cultured with PBS, GelMa, GelMa‐PP, and GelMa‐PP&VEGF scaffolds and fell into four groups. During 3 days of coculturing, the cells were incubated with 1 µl mL^−1^ Calcein‐AM to observe the cell density and morphology under a fluorescence microscope. The CCK8 assay was also applied to measure cell activities in different groups. In addition, the MC3T3‐E1 was seeded on the GelMa and GelMa‐PP scaffold to observe the cell adhesion performance of the scaffold.

### Degradation Test

In order to test the in vitro degradation performance of the scaffold, 0.5 g PP scaffold was incubated in 2.5 ml PBS with 0.1 U mL^−1^ collagenase at 37 °C for 5 weeks. Every week, the PBS with 0.1 U mL^−1^ collagenase was replaced and the PP scaffold was collected and the residual weight was measured after vacuum freeze drying.

### Tube Formation Test

HUVECs were applied to test the angiogenetic ability of the scaffolds. Briefly, different scaffolds including GelMa, GelMa‐PP, and GelMa‐PP&VEGF were cultivated with the cell culture medium for 48 hour to prepare a soaked solution. Subsequently, the 24‐well plates were coated with 200 µl Matrigel for 1 h at 37 °C. Then, HUVECs (≈1 × 10^5^ mL^−1^) in soaked solution were seeded into the Matrigel‐coated 24‐well plates. After 6 h of cultivation, the cells were incubated with 1 µl mL^−1^ Calcein‐AM to observe the tube formation under the light microscope, and the numbers of the formed tubes were counted and recorded.

### Osteogenic Differentiation

The osteogenic activity of the 3D scaffold was investigated via the osteogenic differentiation of BMSCs. Briefly, BMSCs (≈5 × 10^4^ mL^−1^) were seeded into the 0.5% gelatin‐coated cell plates and cultured for 2 days. When the cell grew to ≈80% confluent, different scaffolds including GelMa, GelMa‐PP, and GelMa‐PP&VEGF were put into the upper chamber of the transwell, and the cells were cultured in the osteogenic induction culture medium under chamber for 2 weeks. Then, the cells were immobilized by 4% paraformaldehyde and stained with AR and ALP for osteogenic differentiation characterization. In addion, osteogenesis markers including th RUNX2, ALP, OCN were analyzed with Western blots and RT‐qPCR assay.

### BMSCs Scratch

BMSCs (≈5 × 10^4^ mL^−1^) were plated into a 6‐well plate for scratch assay. When the BMSCs were grown to confluence, the cells were scratched by a 200 µl pipette tip and washed with PBS to remove the unattached cells. Then, transwells with different kinds of scaffolds were put into the cell culture plate. After being treated for 24 h, the cells were stained by calcein‐AM and captured by a fluorescence microscope.

### Hemolytic Test

The rat blood was obtained via a cardiac blood collection method. The collected rat blood was then centrifuged at 2500 rpm for 5 min to separate erythrocytes. Then the supernatant was removed, and the residual was washed with PBS 3 times. After that, the cleaned erythrocyte was diluted to 5% cell suspension by PBS and divided into four groups including water as a positive control, PBS as the control, GelMa, and GelMa‐PP groups. After they were incubated for 4 h at 37 °C, the supernatant was collected by centrifuging and its absorbance was measured at 540 nm.

### In Vivo Bone Regeneration

In a typical experiment, the 5 mm diameter skull defect SD rat model was built to investigate the therapeutic effects of scaffolds. In detail, the rats were anesthetized with 30 mg k^−1^ g pentobarbital sodium. After exposing the surgical field, the skull defect was constructed by a 5 mm diameter ring drill. Then the rats with skull defects were divided into four groups, termed the control, GelMa, GelMa‐PP, and GelMa‐PP&VEGF groups. In the materials treated groups, the round scaffolds of 5 mm diameters were implanted in the defective regions. After 8 weeks of treatment, the rats were sacrificed for further micro‐CT and histological analyses.

### Micro‐CT and Histological Analyses

The collected skull samples were fixed in 4% paraformaldehyde for 1 week. Then, samples were washed 3 times with PBS and scanned by a micro‐CT machine (SkyScan 1176, Bruker, Germany) to evaluate bone regeneration. The scanning resolution was 18 µm and the 3D reconstruction was realized by a Micro‐CT 3D creator software. The BV/TV ratio and BMD were determined via SkyScan software. After scanning, the samples underwent decalcification, and dehydration, and were embedded in the paraffin. Afterwards, they were cut into 5 µm slices and stained with H&E, Masson's, CD31, OPN, and OCN for further evaluation. In addition, the heart, liver, spleen, lung, and kidney were collected for H&E staining.

### Statistical Analysis

All data were expressed as means ± standard deviations (n≥3). One‐way ANOVA was applied to analyze the difference in independent groups. The statistical significance was defined at the value of *p < 0.05, **p <0.01, and ***p < 0.001.

## Conflict of Interest

The authors declare no conflict of interest.

## Author Contributions

Y.J.Z. conceived the conceptualization and designed the experiment. L.Y. carried out the experiments. L.F., X.L., and Y.R.Y. participated in data analysis and discussion. Y.R.Y. and L.Y. wrote the paper.

## Supporting information

Supporting Information

## Data Availability

The data that support the findings of this study are available in the supplementary material of this article.
