# Supplementary material for: Pearl Powder Hybrid Bioactive Scaffolds from Microfluidic 3D Printing for Bone Regeneration
Source: Adv Sci (Weinh). 2023 Oct 23;10(34):2304190. doi: 10.1002/advs.202304190 (PMC10700190; doi:10.1002/advs.202304190)
Supplement: Supplementary file 1 — Supporting Information [file ADVS-10-2304190-s001.pdf]

ADVANCED SCIENCE
Open Access

Supporting Information

for *Adv. Sci.*, DOI 10.1002/advs.202304190

Pearl Powder Hybrid Bioactive Scaffolds from Microfluidic 3D Printing for Bone Regeneration

Lei Yang, Lu Fan, Xiang Lin, Yunru Yu and Yuanjin Zhao**

Supporting Information

Pearl Powder Hybrid Bioactive Scaffolds from Microfluidic 3D Printing for Bone Regeneration

Lei Yang, Lu Fan, Xiang Lin, Yunru Yu*, Yuanjin Zhao*

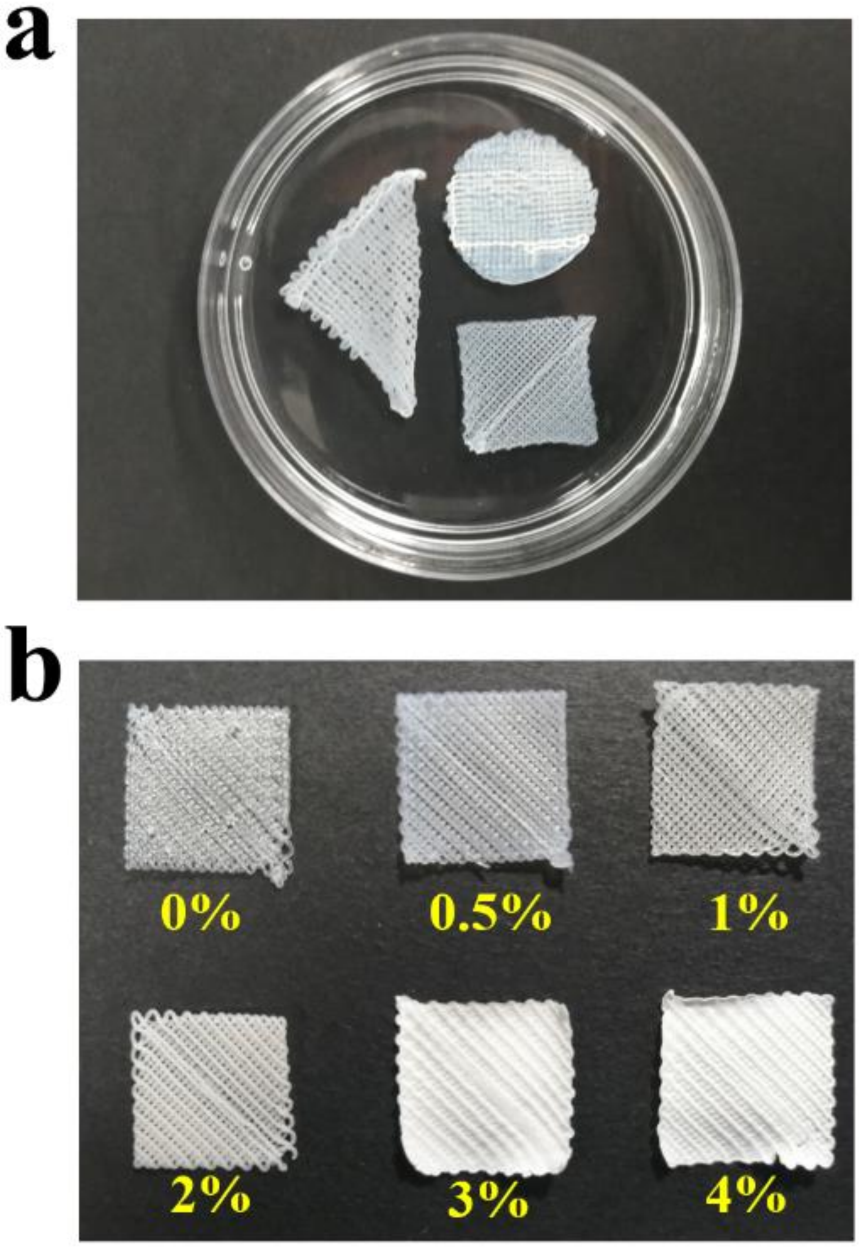

Figure S1. PP hybrid scaffolds with (a) different shapes and (b) PP concentrations.

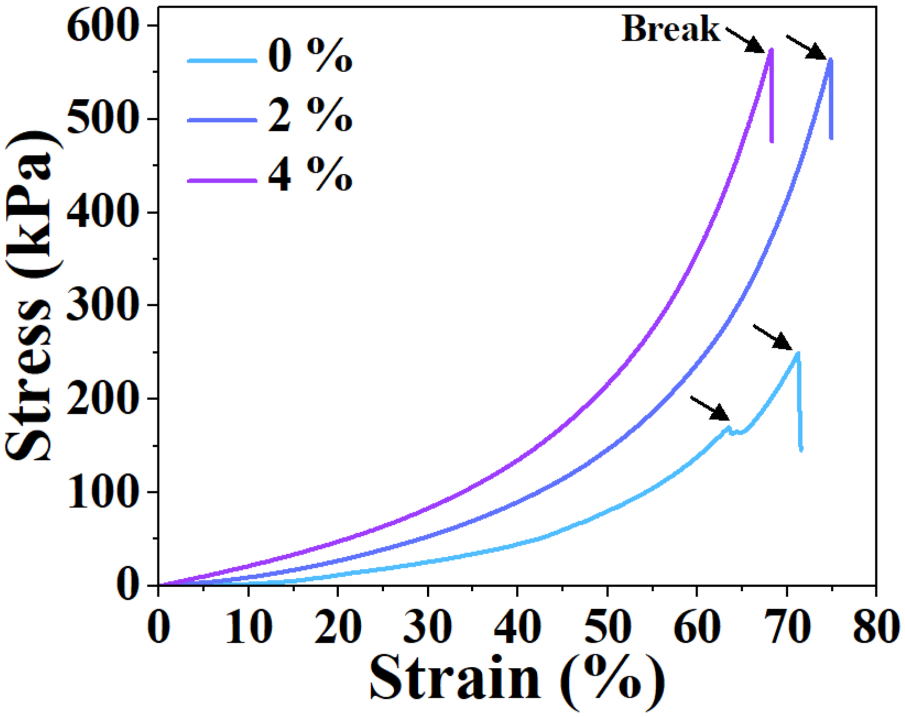

Figure S2. The mechanical strength of hybrid scaffolds with different PP concentrations.

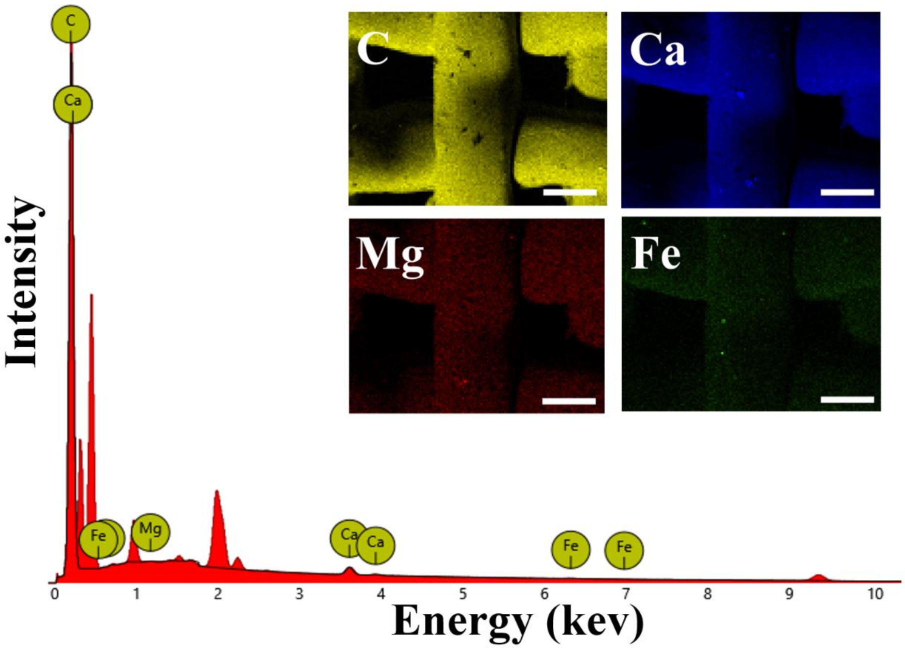

Figure S3. Element spectrum of the PP scaffold including C, Ca, Mg and Fe. Scale bars are 200 μm.

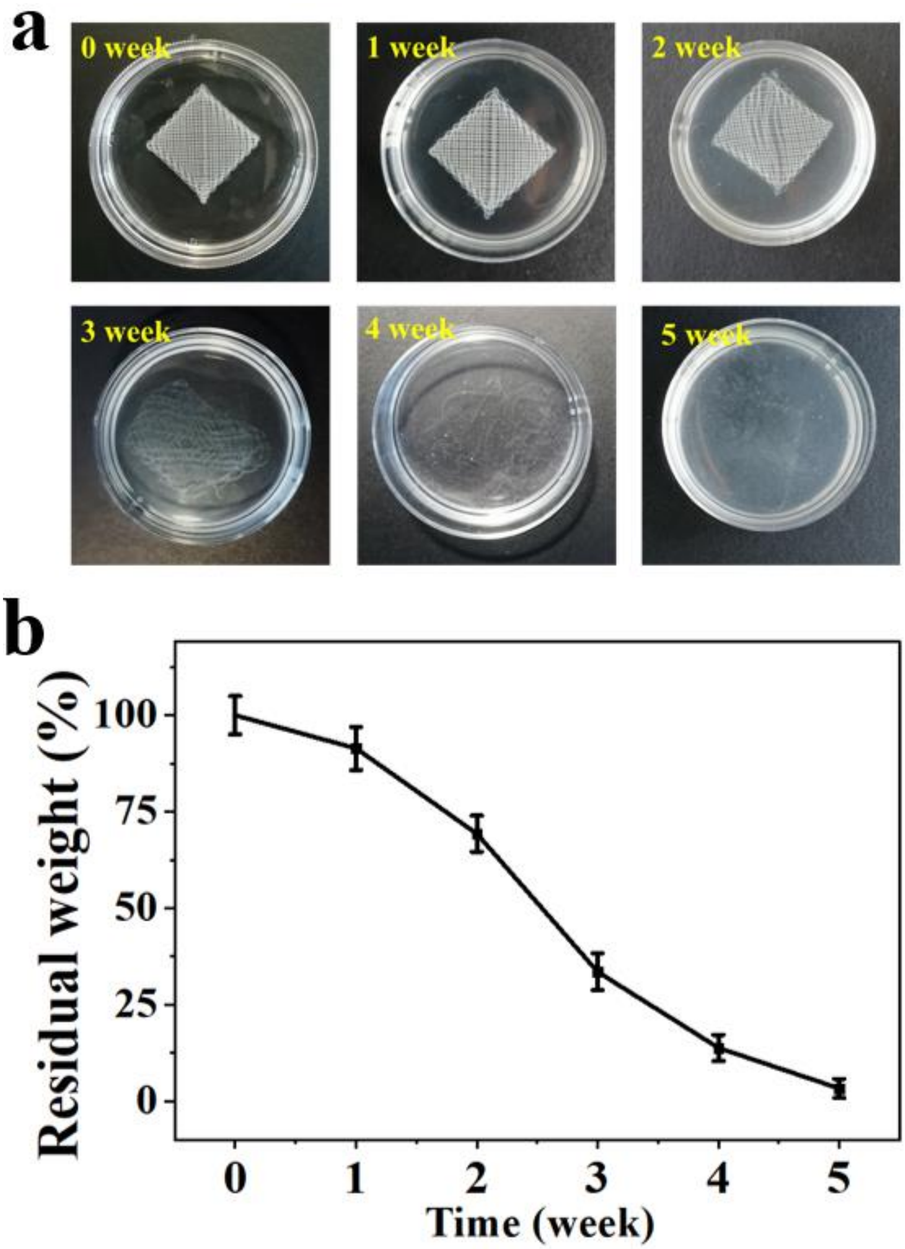

Figure S4. In vitro degradation study of PP scaffolds over 5 weeks. (a) Digital images of PP scaffolds over 5 weeks. (b) The residual weight of PP scaffolds over 5 weeks. Data (n≥3) are shown as mean ± SD.

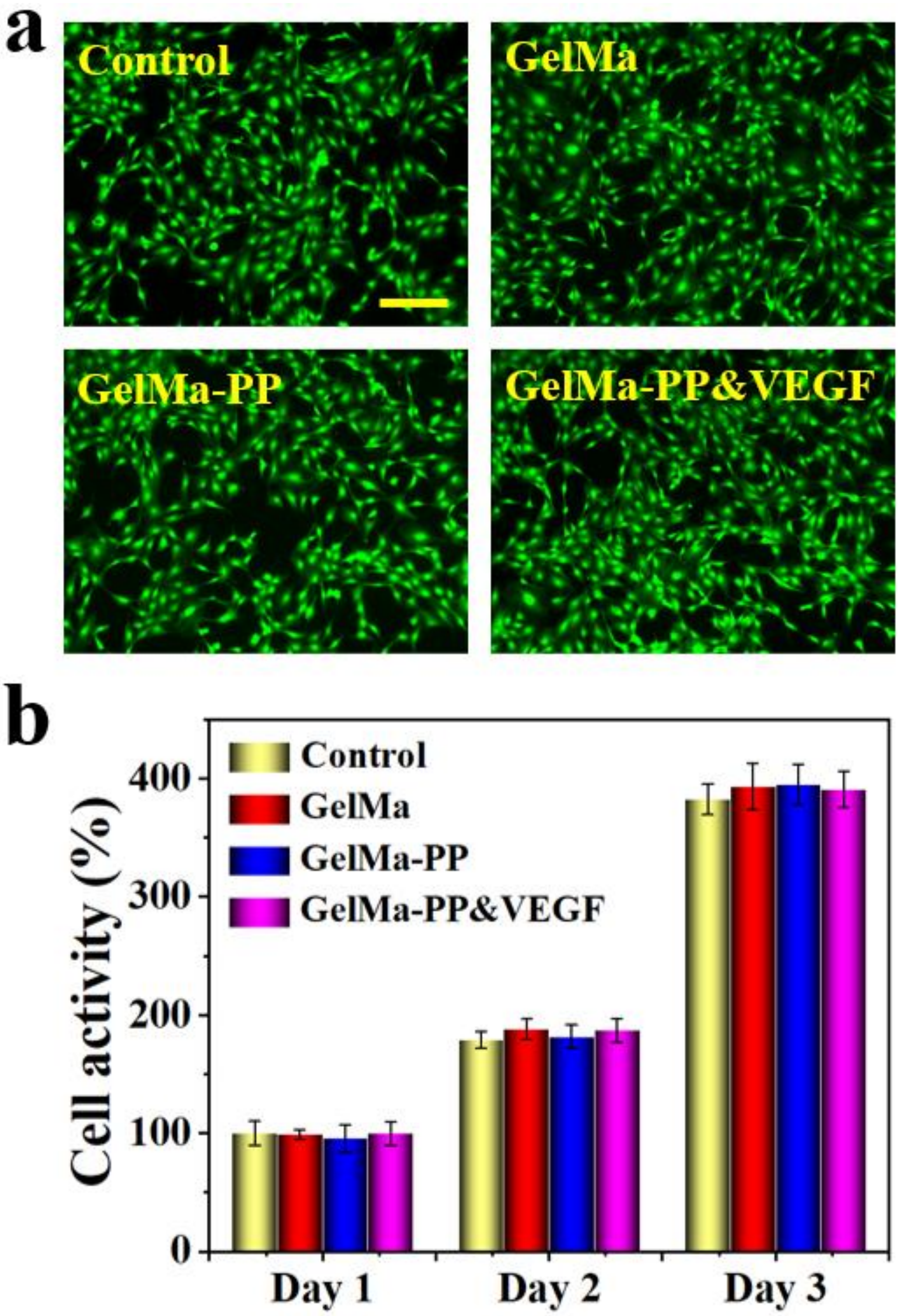

Figure S5. Cell morphologies (a) and cell activity (b) of MC3T3-E1 cells for 3 days. Scale bar is 200 μm. Data (n≥3) are shown as mean ± SD.

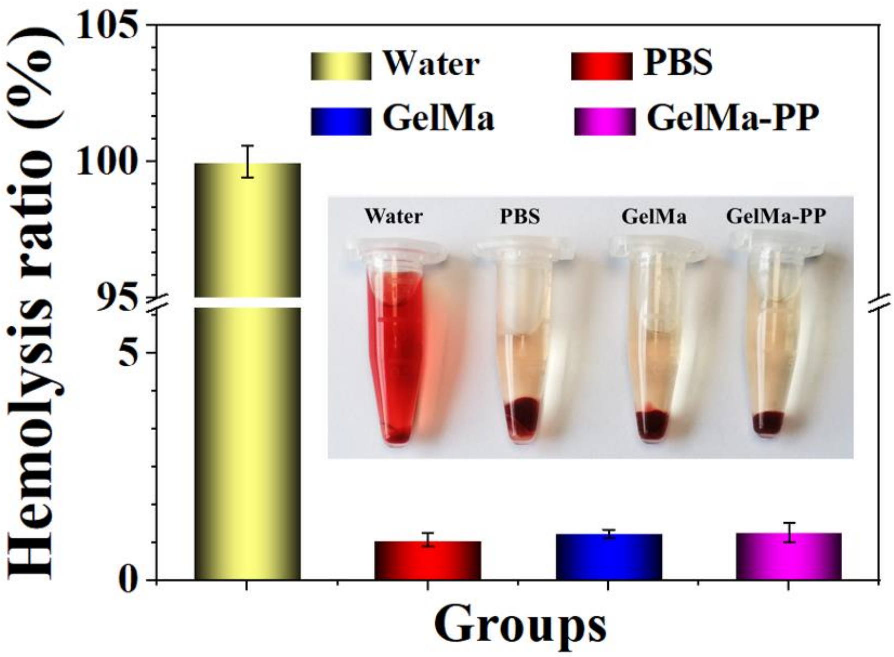

Figure S6. Hemolysis assay for 3D scaffolds with or without PP. Data (n≥3) are shown as mean ± SD.

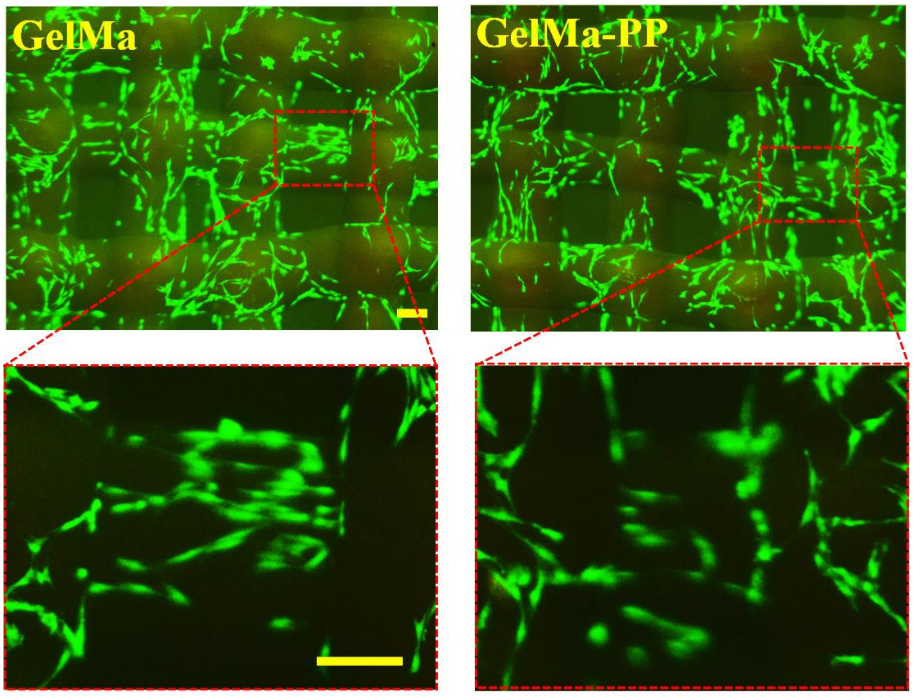

Figure S7. Fluorescent images of cells adhered on the GelMa and GelMa-PP scaffolds. Scale bar is 100 μm.

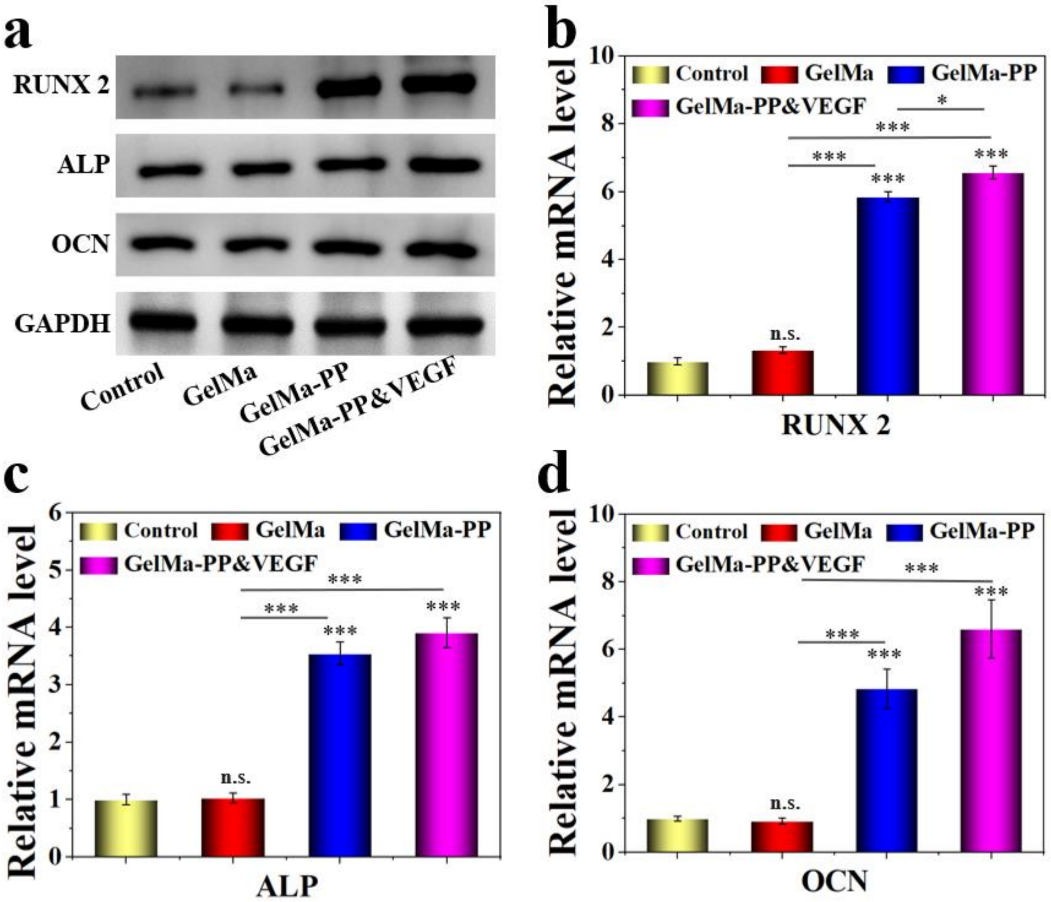

Figure S8. The osteogenic markers expression o in vitro. (a) The protein expression levels of osteogenic markers including RUNX2, ALP and OCN. (b-d) The mRNA expression of osteogenic markers including (b) RUNX2, (d) ALP and (d) OCN. Data (n≥3) are shown as mean ± SD. n.s.: no significant, * $p < 0.05$, ** $p < 0.01$, ***$p < 0.001$.

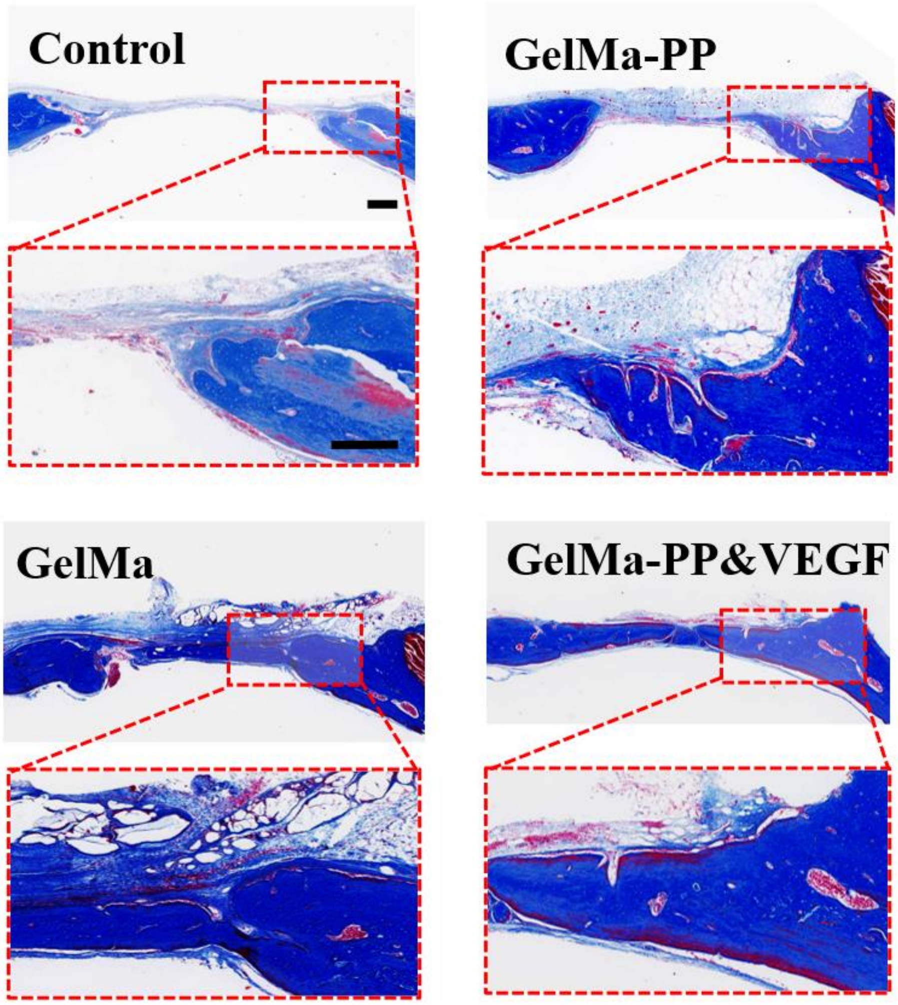

Figure S9. Masson's trichrome staining images of different groups. Scale bar is 500 μm.

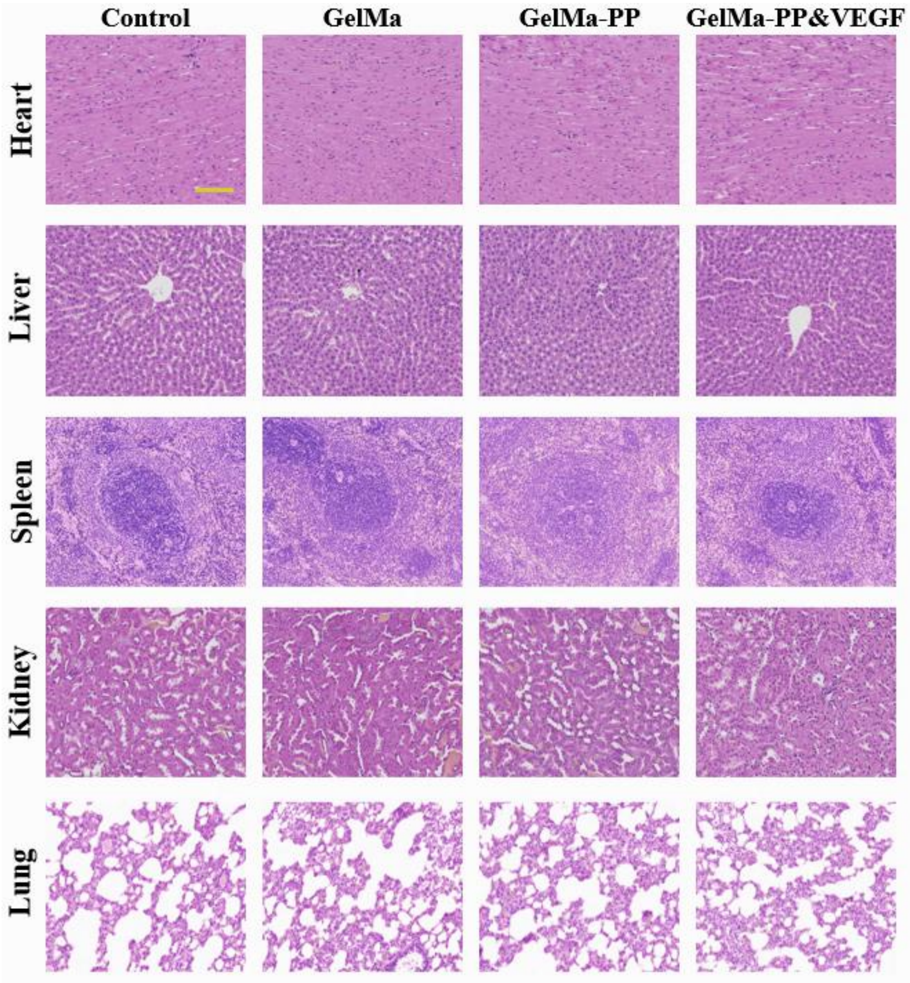

Figure S10. H&E staining images of the heart, liver, spleen, lung, and kidney of rats. The scale bar is 100 μm.
